# Beam Offset Detection in Laser Stake Welding of Tee Joints Using Machine Learning and Spectrometer Measurements

**DOI:** 10.3390/s22103881

**Published:** 2022-05-20

**Authors:** Aydin Jadidi, Yongcui Mi, Fredrik Sikström, Morgan Nilsen, Antonio Ancona

**Affiliations:** 1 Department of Engineering Science, University West, 461-32 Trollhättan, Sweden; yongcui.mi@hv.se (Y.M.); fredrik.sikstrom@hv.se (F.S.); morgan.nilsen@hv.se (M.N.); antonio.ancona@hv.se (A.A.); 2 Physics Department, University of Bari, Via Orabona 4, 70126 Bari, Italy

**Keywords:** laser beam offset, feature selection, laser beam welding, machine learning, spectrometer, tee joint

## Abstract

Laser beam welding offers high productivity and relatively low heat input and is one key enabler for efficient manufacturing of sandwich constructions. However, the process is sensitive to how the laser beam is positioned with regards to the joint, and even a small deviation of the laser beam from the correct joint position (beam offset) can cause severe defects in the produced part. With tee joints, the joint is not visible from top side, therefore traditional seam tracking methods are not applicable since they rely on visual information of the joint. Hence, there is a need for a monitoring system that can give early detection of beam offsets and stop the process to avoid defects and reduce scrap. In this paper, a monitoring system using a spectrometer is suggested and the aim is to find correlations between the spectral emissions from the process and beam offsets. The spectrometer produces high dimensional data and it is not obvious how this is related to the beam offsets. A machine learning approach is therefore suggested to find these correlations. A multi-layer perceptron neural network (MLPNN), support vector machine (SVM), learning vector quantization (LVQ), logistic regression (LR), decision tree (DT) and random forest (RF) were evaluated as classifiers. Feature selection by using random forest and non-dominated sorting genetic algorithm II (NSGAII) was applied before feeding the data to the classifiers and the obtained results of the classifiers are compared subsequently. After testing different offsets, an accuracy of 94% was achieved for real-time detection of the laser beam deviations greater than 0.9 mm from the joint center-line.

## 1. Introduction

Laser beam welding (LBW) is an important manufacturing process with a widespread application from automotive and ship building to aerospace and micro-electronics [1,2]. LBW presents many advantages compared to traditional arc welding such as higher welding travel speeds, ease of automation, less heat induced residual stress and deformation, deeper penetration and forming a small heat affected zone [3,4,5,6]. In addition, being a non-contact method, LBW offers extreme flexibility in the geometry of the joint to be welded such as in the case of tee joints. Tee joints provide higher structural strength and lower weight, and stake welded tee joints have wide applications for example in marine industry [7,8,9].

However, just like other manufacturing- and welding processes, defects and discontinuities may be formed during LBW. Some of the defects can be prevented or corrected by in-process monitoring and control. Furthermore, collected data during the process, can be used for offline analysis or building machine learning (ML) models to be used in-process applications such as go/no-go systems.

In-process monitoring of LBW has been subject to extensive research, and different combinations of sensors and algorithms have been used to detect defects and discontinuities. Considering the characteristics of LBW, most of the monitoring methods are based on data from optical radiations from the welding zone [10]. However, acoustical sensors and X-ray imaging are also used for monitoring LBW [11,12,13,14,15]. Monitoring methods are developed based on the target characteristic such as joint gap width variations, spatter, keyhole stability and laser beam (LB) offset detection. A high-power LBW monitoring system was developed by Li et al. [16], using X-ray transmission imaging system and high speed cameras. The research investigated the relation between the molten pool behavior and formation of spatters. Another monitoring system was developed in [17] for dynamic keyhole profile monitoring based on the vapor-generated wave.

In another research [18] the correlation analysis of the plasma plume optical spectra generated during LBW was used for in-process monitoring. As a result, the formation of weld defects were detected using the optical emissions and the co-variance mapping technique. The laser-induced plasma plume electron temperature signal, calculated based on the optical spectra acquired during the LBW process, was also shown in [19] to be correlated to the occurrence of welding defect. Sibillano et al. [20] utilized an optical spectroscopic sensor for real-time control of the LBW process. The optical emission above the keyhole was used to calculate the laser plasma plume electron temperature. The relationship between the electron temperature and penetration depth was used as the input to a PI controller with the purpose of stabilizing the penetration depth in overlap welding. The results were validated afterwards using microscopic analysis of the seam cross sections.

In addition to the spectrometers for real-time and non-destructive monitoring, photo-diodes also have been utilized for monitoring of LBW [21,22,23]. However, a combination of photo-diodes is necessary to cover the whole relevant spectral range. In a study conducted by Nilsen et al. [24], a dual vision and spectroscopic sensing system was employed to monitor a varying joint gap during LBW. Image processing techniques followed by Kalman filter were used to estimate the gap width, using the data captured by a CMOS camera. In addition, the gap estimation was realized using the intensity of spectral lines provided by a spectrometer.

Machine learning techniques are widely used for classification and prediction problems in diverse fields [25,26,27,28,29,30,31,32] as well as welding monitoring and discontinuity detection. In a research conducted by Chen et al. [33] artificial neural network (ANN) and support vector machine (SVM) were employed to classify welding defects using emission spectrum data. Feature extraction methods were applied before feeding data to the classifiers. The data were divided into 3 labels, namely no defects, pores and bead separation. The results indicated good performance of the classifiers in detecting defects and ANN demonstrated slightly better results compared to SVM.

In another research [34], a deep neural network (DNN) was employed for quality assessment of the LBW. A spectrometer used for collecting data and and the measured data were converted to RGB values. The collected data were related to 4 classes of unwelded, incomplete penetration, full penetration and unwelded with a gap. After training the model, a classification accuracy of 90% was achieved. Fan et al. [35] developed a model based on auxiliary classifier generative adversarial network (ACGAN) and convolutional neural networks (CNN), for real-time LBW defect detection. ACGAN was used to generate fake data based on data collected by optical and thermal sensors, to increase the samples in training data set, and CNN classified the data.

**Figure 1:**
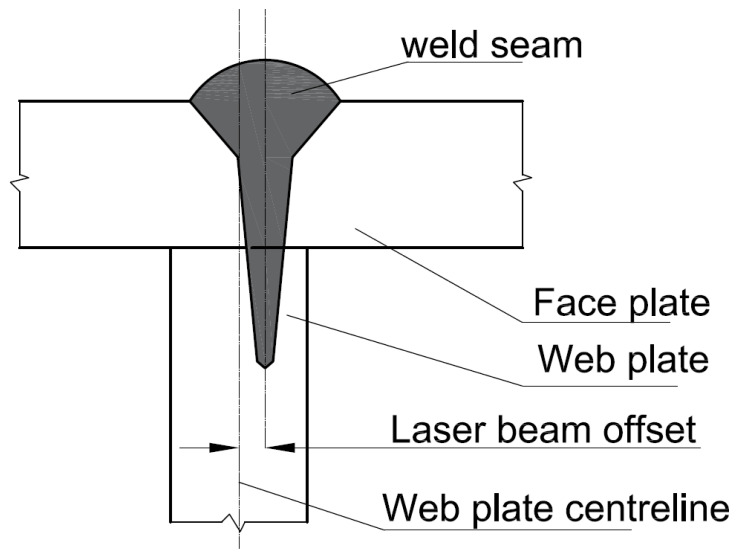
Stake welded tee joints [36].

On-line beam offset detection is particularly relevant for tee joints. Indeed, as seen in Figure 1, during welding of tee joints, it is not possible to detect the deviation (beam offset) between the LB and the joint center-line from the top side by optical means, due to lack of visibility. Beam offset detection during LBW has also been subject of several studies. In a method developed in [36], a photo-detector and change point detection methods were used for on-line and off-line LB deviation detection. In another study using photodiode and signal processing [37], laser beam deviations were detected approximately 10 mm and 15 mm after the movement out of the joint using offline and online methods respectively.

In addition to the mentioned signal processing methods for LB offset detection, a neural network was employed to detect the LB deviations from the join center line using images collected by a camera and feature extraction techniques [38]. The extracted features from the vision camera were used as inputs of a nonlinear autoregressive model. However, error indicator results are not provided.

Despite previous and on-going research in LBW monitoring, lack of studies related to LB deviation detection using ML, motivated the current study. This work fills this gap and focuses on the monitoring of LBW of the tee joints for automatic LB offset detection. A spectrometer was used for collecting the spectral emissions from the laser-metal interaction zone and different classifiers were tested to investigate their provided classification accuracy for the data. Before feeding the collected data to the classifiers, feature selection was applied to select discrete wavelengths of the spectrometer, to increase the classification accuracy and reduce the size of input vector. The equipment used in experiments is described in Section 2, as well as data and data pre-processing steps and information about the classifiers. The results are presented in Section 3 and are discussed in Section 4. Finally, Section 5 is the conclusion.

## 2. Materials and Methods

### 2.1. Welding Setup and Spectrometer Measurements

**Figure 2:**
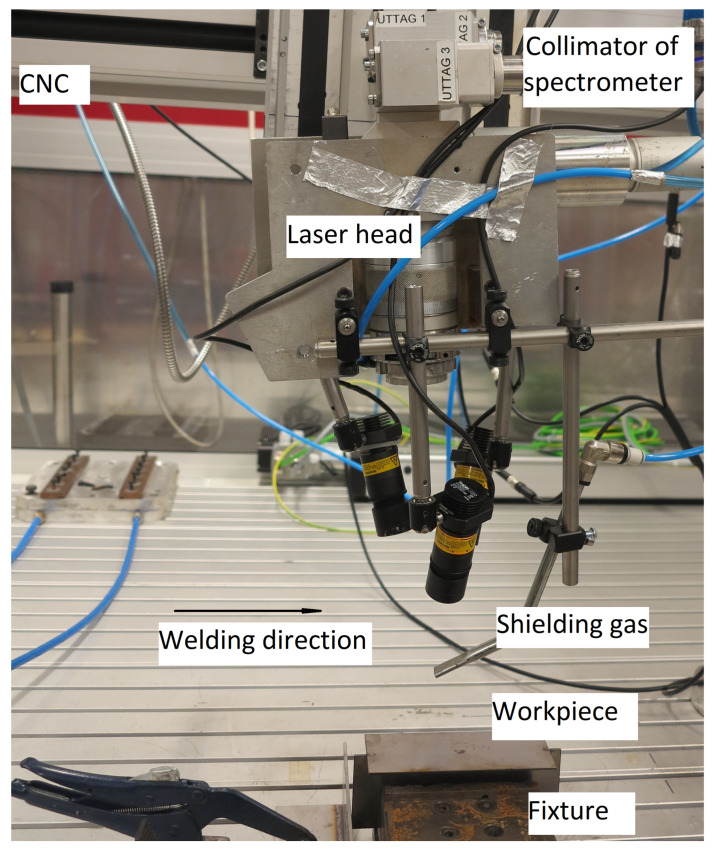
The welding setup for the experiments.

The LBW equipment consists of a CNC gantry, a water-cooled high power ytterbium-doped fiber laser and fixturing. The CNC gantry (from Isel^®^ Germany mod. M40) was used to maneuver the welding tool from Permanova Lasersystem AB. The LB with a Rayleigh length of 12.5 mm was generated by a 6 kW IPG laser system (mod. YRL-6000-S) with a 1070 nm wavelength. The LB was focused on the face plate top surface with a diameter of 1.5 mm in focus and the power was in continuous wave resulting in keyhole mode welding. The fixed power was set at 3500 W, the welding travel speed was 15 mm/s, and the flow rate of the shielding gas Ar was 20 L/min. These parameters were experimentally derived to produce a visibly good-looking weld seam. The welding was conducted without filler material, and the plate material was S355MC high strength steel for both face and web plates. As illustrated in Figure 2, the thickness of the plates was 2 mm and the width was 60 mm. The lengths of the face and the web plates were 150 mm and 170 mm, respectively. Figure 2 shows a reference welding and different welding cases with linear deviations of the LB from the web plate center line.

The welding was monitored by a spectrometer (Ocean Optics HR2000+). During welding, hot vapour emerging from the keyhole which is weakly ionized and a plasma plume is formed. The generation of such plasma plume is frequently observed also in case of 1-um wavelengths lasers, where the beam quality is high and multi-kW power is employed producing a high laser intensity on the metal surfaces. The atomic and weakly ionized chemical species composing such plasma plume, emit optical radiation that can be collected by optical sensors such as spectrometers.

Compared to photo-diodes, spectrometers can resolve the incoming signal and separate each wavelength, providing the possibility to get more information about the state of the process, such as the plasma electron temperature [39,40]. In addition, the high dimension of discrete vector data acquired by the spectrometer enables the usage of advanced signal analysis methods, such as ML. The spectrometer is low-cost and can be easily implemented in a welding tool since it is a compact and non-contact device. The spectrometer used in this work, is equipped with a 2048 pixel CCD array detector and 10 µm entrance slit. It collects light in the wavelength range between 400–530 nm with a spectral resolution of 0.07 nm. The welding setup for the experiments is presented in Figure 2.

**Figure 3:**
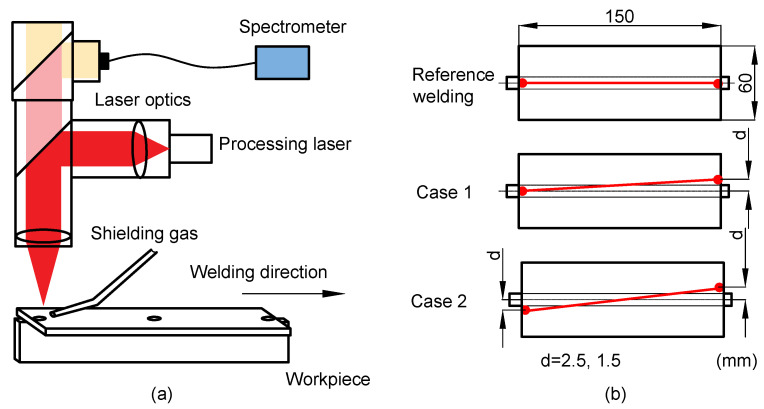
The welding setup and controlled beam deviations. (**a**): Welding setup; (**b**): Top-view of the joint. The red line indicates the beam path with controlled deviation.

The spectrometer was connected to the welding tool and used to collect light from the interaction zone which was sent to the spectrometer entrance slit through a 200-μm core optical fiber, as illustrated in Figure 3a. The exposure time during the acquisitions was 20 ms.

### 2.2. Data Collection and Pre-Processing

**Figure 4:**
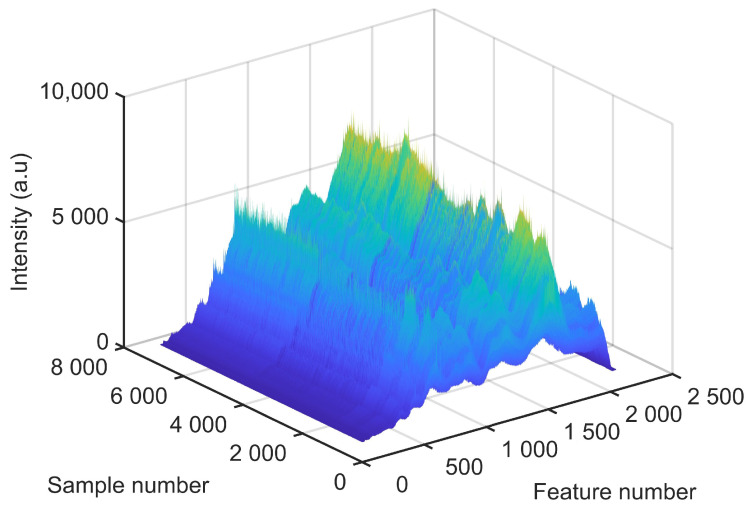
Time series of spectral data acquired during the tests. Each feature represents discrete wavelengths of the spectrum while the sample number represents the time sequence acquired during the tests.

In order to collect data, 14 different tests were realized where, in 4 reference test cases, the LB spot followed the straight centre line of the joint, and in the other test cases controlled linear deviations were applied (as seen in Figure 2). The collected raw data related to all test cases are presented in Figure 4.

As the next step, data of 2 tests related to controlled linear LB deviations with an offset between −1.5 mm to 1.5 mm and −2.5 mm to 2.5 mm were separated to be the testing data sets of the classifiers. The remaining data were used for feature selection and training of the classifiers. The number of samples collected from each test were divided by the LB deviation range, and the obtained numbers were set to be class 1 (without deviation) or class 2 (with deviation), according to the pre-determined LB deviation limit. Afterwards the labeled samples were shuffled and in order to balance the data in class 1 and class 2, the same sample number of each class were selected.

Random forest algorithm was used for selecting important wavelengths and in addition, a non-dominated sorting genetic algorithm II (NSGA II) was employed for feature (wavelengths) selection and its cost function was set to be the mean accuracy for the testing data set of an multi-layer perceptron neural network (MLPNN) in 10 runs, for each individual in each iteration. Data division for testing and training data sets of the cost function, was set to be random (80% for training and 20% for validating and testing). The testing data set for each individual in each iteration was unknown for the MLPNN, employed as the cost function.

The selected wavelengths were fed to the classifiers namely MLPNN, support vector machine (SVM), learning vector quantization (LVQ), logistic regression (LR), decision tree (DT) and random forest (RT) and the obtained results from classifiers in each step of the process were reported in Section 3.

#### Non-Dominated Sorting Genetic Algorithm II (NSGA II)

Non-dominated Sorting Genetic Algorithm II is a multi-objective optimization algorithm, and it starts with a temporary solution in each iteration ($R_{t}$) containing the population ($P_{t}$) and the offspring population of the same size ($Q_{t}$) [41]. The offspring population is generated by using genetic operations of mutation and crossover. It merges the parents and offspring population and as a result, the next generation contains the best individuals from both parents and offspring space. As a result, the best individuals will be kept during the evolution process (elitism) [42]. After combining the parents and offspring population, the population is ranked. Individuals in the first front, are not dominated by the other individuals, and the individuals and consequent fronts are only dominated by the individuals in previous fronts. The selection of parents for generating the next population ($P_{t+1}$) is based on the number of the front and calculating the distance of each population individual with the neighboring individuals in the same front by using crowded-comparison operator [43]. The crowding distance indicates the density of solutions surrounding an individual. In order to maintain the diversity, individuals in less crowded regions are preferred [44].

### 2.3. Classifiers

#### 2.3.1. Multi-Layer Perceptron Neural Network (MLPNN)

Multi-layer perceptron neural networks consist of input layer, at least one hidden layer and output layer. The MLPNN employed in this work, is a three layers feed-forward network, in which all neurons of the previous layer are connected to the all neurons of the next layer. The weights of these connections are updated during the training process. The output of each neuron is multiplied by the connection weight and passes through an activation function and summation with the outputs of the other connected neurons [45]. The output of each layer is calculated by following equation:

(1) $$a=\phi (\sum_{j}^{}w_{j}x_{j}+b)$$

where, $w_{j}$, $x_{j}$, *b* and ϕ are weights, inputs, bias and activation function respectively.

#### 2.3.2. Support Vector Machine (SVM)

Support vector machine is a supervised learning method introduced by Boser, Guyon, and Vapnik [46]. It is one of the most used classification methods because of its robustness and generalization ability [47]. SVM separates the data by matching them to feature space and using a hyper-plane and if data are not linearly separable, nonlinear kernels are used [48]. The common mathematical description of this optimization problem is given below:

(2) $$\begin{array}{c} \min_{\omega ,b,\xi_{i}}\frac{1}{2}\omega^{T}\omega +C\sum_{i}^{}\xi_{i}\\ s.t.y_{i}\left(\omega^{T}\phi \left(x_{i}\right)+b\right)\geq 1-\xi_{i},\xi_{i}\geq 0 \end{array}$$

where, *w*, φ, $x_{i}$, $y_{i}$, *b* are normal vector, transformation function, point on the hyper-plane described by the normal vector, labels and offset of the normal vector from the beginning respectively. *C* is the penalty coefficient and the radial basis function (RBF) kernel is defined as:

(3) $$\begin{array}{c} \phi {\left(x_{i}\right)}^{T}\phi \left(x_{j}\right)=exp\left(-\gamma {∥x_{i}-x_{j}∥}_{2}^{2}\right) \end{array}$$

where, γ is a positive hyperparameter.

#### 2.3.3. Learning Vector Quantization (LVQ)

Vector quantization theory is a controlled version of Kohonen network also known as self organizing maps (SOM). SOM is an unsupervised learning algorithm based on mapping a high dimensional input to a feature space (grid) [49]. LVQ is a supervised classification neural network algorithm based on SOM which finds a set of prototype vectors (codebook vectors) that represent the input space and the input data are processed based on their similarity to the prototype vectors [50]. If the processed data is from the same class as the prototype vector, the vector is moved towards the training sample (winner takes all) [51], and if it is not from the same class, the prototype vector moves apart. After evaluating all prototype vectors against all training samples in the feature space, the updated prototype vectors are used to assign classes for the unseen data. The process is similar to the Lloyd’s algorithm, but the codebook vectors are used for making the prediction.

#### 2.3.4. Random Forest (RF)

**Figure 5:**
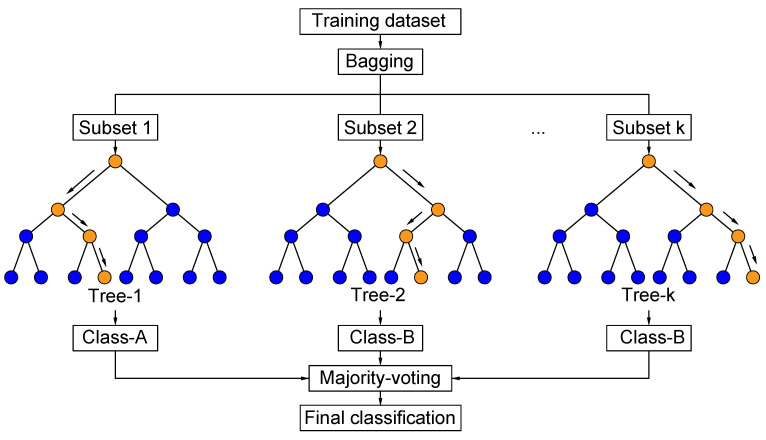
Flowchart of RF as a classifier [54].

Random forest is an ensemble learning model and consists of multiple decision trees. When a new input sample is fed to the algorithm, all decision trees classify independently, and the final classification result is obtained by the majority voting as illustrated in Figure 5. RF uses sampled training set to train each DT and the randomized training set is extracted by using Bootstrap method. Using a set of decision trees makes the RF resistant to overfitting and no pruning for the DT is needed because of random relation between sub-samples [52,53].

## 3. Classification Results

Different linear deviations from the joint center were used to test the accuracy of the employed classifiers. The accuracy in each case are presented in Table 1. The reported values are the median accuracy of 10 runs of each classifier and all wavelength has been used as the inputs. A definition of the error indicators can be found in Appendix A.

**Figure 6:**
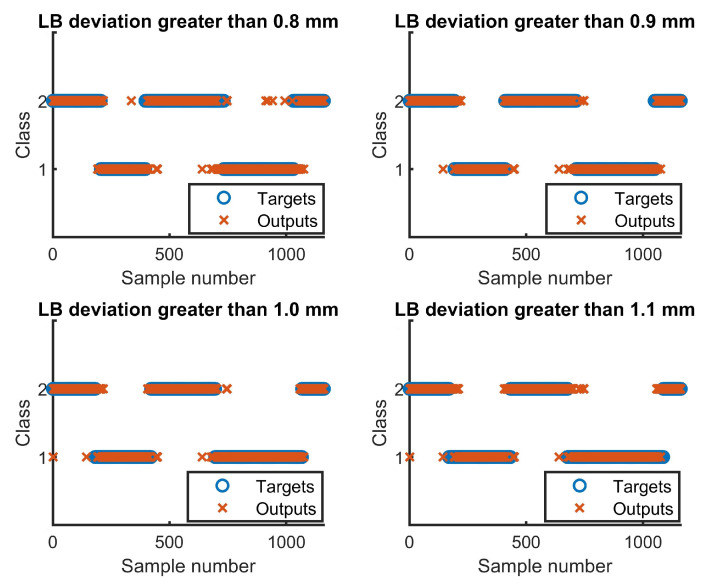
Outputs and targets for detecting LB deviations larger than 0.8 mm, 0.9 mm, 1 mm and 1.1 mm.

The MLPNNs employed in this research have only one hidden layer. Networks with more than one hidden layer have been tested and did not provided higher accuracy. Two data sets with linear offset between −2.5 mm to +2.5 mm and −1.5 mm to +1.5 mm were used as testing data set in all reported results in Table 1. Minus and plus stand for beam offset deviation from the left side and to the right side of the joint center line respectively. As seen, using a LB deviation of 0.9 mm for classification, provides higher classification accuracy, compared to other tested LB deviations. Outputs and targets for detecting LB deviations larger than 0.8 mm, 0.9 mm, 1 mm and 1.1 mm is presented in Figure 6.

**Figure 7:**
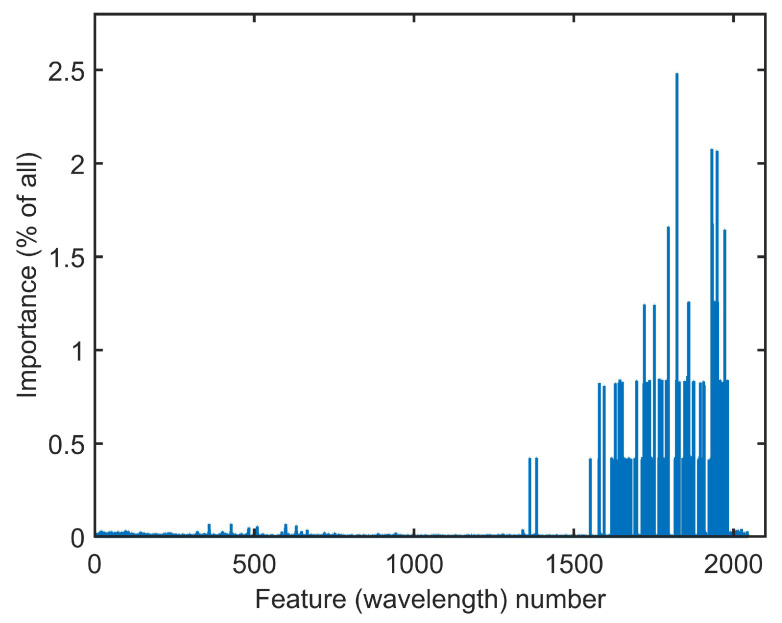
Spectrometer feature (wavelength) importances obtained by RF.

The results reported in Table 1 are generated using all wavelengths of the spectrometer. However, it is possible to obtain the importances of the input features, which sum to one, by using random forest algorithm. The importance value for each feature is the average of its importances over all trees and are shown in Figure 7.

Among the importances obtained for the features using RF, 127 of them have importance greater than 0.4% and they were used as the inputs for the classifiers. The results are given in Table 2 and are related to the median accuracy of 10 runs for each classifier.

**Figure 8:**
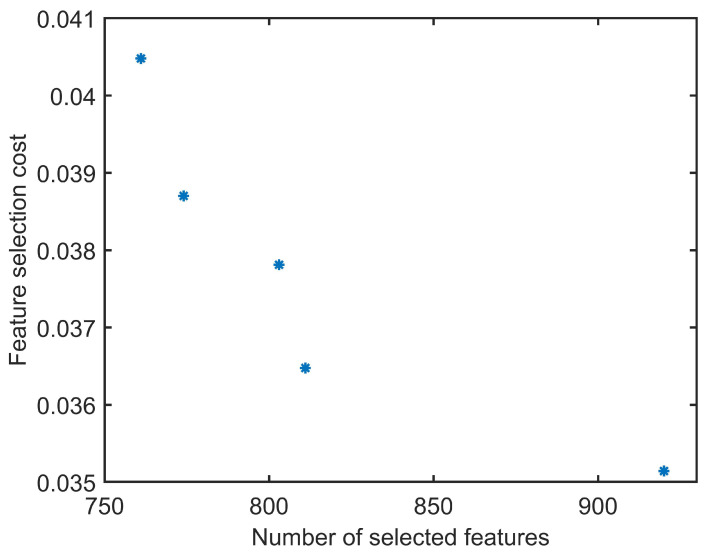
Pareto-front of the NSGA II.

As the next step, the NSGA II was used to define the input set of the classifiers. The output of this algorithm is not only one set of inputs. NSGA II provides non-dominated results in its Pareto-front which, all of them can be used based on the computational power and the desired accuracy. The Pareto-front of the NSGA II in current work, is shown in Figure 8, which describes the feature selection cost for the solutions provided by the NSGA II. An MLPNN was used as the cost function of the algorithm. The deviation from the joint center-line was set to 0.9 mm, as according to Table 1, the best accuracy is obtained using a deviation of 0.9 mm. All data was divided randomly to training and testing data sets, in each iteration for each individual. Cost was set to median accuracy of 10 runs for testing the data set.

The classification results related to the set of wavelengths providing higher accuracy (920 wavelengths) are presented in Table 3. Reported results for each classifier are related to the median accuracy of 10 runs for each classifier. Testing data set used in Table 3 is the same that was used to generate the results reported in Table 1 and Table 2.

**Figure 9:**
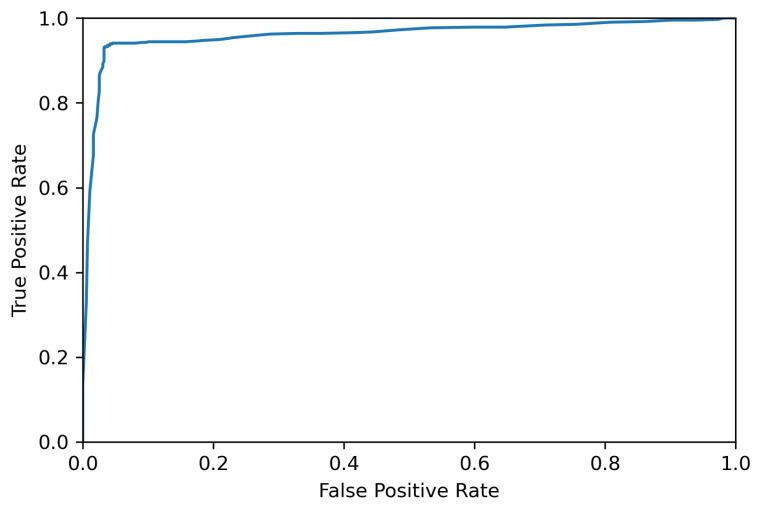
ROC curve of the results from the RF algorithm.

**Figure 10:**
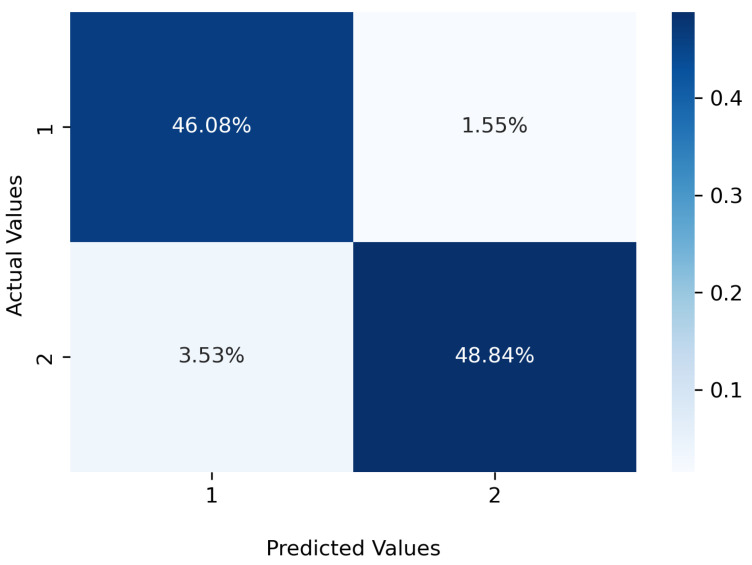
Confusion matrix for the RF algorithm for detecting LB deviations greater than 0.9 mm.

According to Table 2 and Table 3, not all wavelengths are needed to be used as the inputs of the classifiers and the selected set of the wavelengths provides the same or slightly higher accuracy for the selected testing data set. The corresponding receiver operating characteristic curve (ROC) and confusion matrix for RF using 920 selected wavelengths for detecting a deviation of 0.9 mm are presented in Figure 9 and Figure 10, respectively.

**Figure 11:**
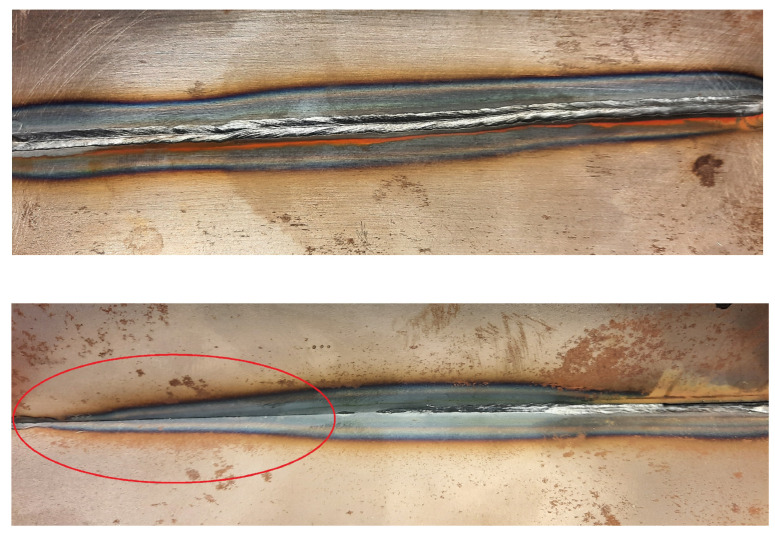
Top and root view of a weld seam with LB deviation from −2.5 mm to 2.5 mm. Lack of fusion between the plates is indicated by red in the root view.

Photos from of the top and root view of a weld seam with controlled LB deviation from −2.5 mm to 2.5 mm is presented in Figure 11 showing typical lack of fusion defects caused by a LB offset. Lack of fusion caused by the LB deviation is marked with red in the root view of the welded plates.

## 4. Discussion

Different LB offset thresholds were tested and for all employed classifiers, detecting the deviations larger than 0.9 mm provided higher classification accuracy (Table 1). This is explained by the underlying physical phenomena and is related to the thickness of the web plate (2 mm) and LB shape with diameter of 1.5 mm. In LB deviations larger than 0.9 mm, melt flow and keyhole are influenced due to the proximity of LB to the edge of the web plate. This has a practical significance since a LB offset of 0.9 mm has a very high probability to result in deteriorating lack of fusion defects.

After training the classifiers, the classifiers generate result for new data very fast and without computational complexity and the trained classifiers can be used as a part an online control system. Most of the misclassifications occurred while the LB offset varies between pre-determined classes (Figure 4). Since each sample represents a very small advance of the LB in welding direction (approximately 0.25 mm), and considering the possibility of misclassification, an online go/no-go system can be programmed to pause the process in case of a few consecutive offset detections.

## 5. Conclusions

In this work, ML techniques were applied for detecting LB deviations from 0.5 mm to 1.3 mm from the joint center-line. The most accurate classification result was obtained in detecting a LB deviation of 0.9 mm. Feature selection using RF and NSGA II was applied to reduce the number of features (wavelengths) in the input vector, without reduction in the classification accuracy.

Not all features of the original data are necessary for detecting the LB deviation, and using selected wavelengths results in a slightly better classification performance, while reducing the number of input features. After applying the proposed methodology, the classification accuracy reached 94% for the testing data. The suggested method has a promising potential to be used in-process as a go/no-go system, to prevent discontinuities such as lack of fusion.

The method can probably also be applied to other types of welding joints especially when the non-visibility condition of the joint center-line exists and it can be expanded to detect other discontinuities during welding.

## Figures and Tables

**Table 1:** Classification accuracy at different LB deviations from the joint center line.

Classifier	Deviation (mm)								
	0.5	0.6	0.7	0.8	0.9	1.0	1.1	1.2	1.3
MLPNN	0.7717	0.8243	0.8686	0.9117	0.9475	0.9410	0.8984	0.8488	0.8084
SVM (RBF)	0.7653	0.8213	0.8742	0.9121	0.9483	0.9422	0.8984	0.8497	0.8144
LVQ	0.7661	0.8165	0.8656	0.9397	0.9436	0.9380	0.8975	0.8463	0.8079
LR	0.8105	0.8337	0.8587	0.8828	0.9138	0.9035	0.8725	0.8260	0.8111
DT	0.7614	0.8173	0.8509	0.8888	0.9267	0.9310	0.8845	0.8527	0.8070
RF	0.7665	0.8204	0.8742	0.9117	0.9483	0.9401	0.8975	0.8570	0.8182

**Table 2:** Classification results for RF selected set of wavelengths.

Classifier	Class	Accuracy	Sensitivity	Specificity	Precision	F-Score
MLPNN	1	0.9475	0.9675	0.9293	0.9256	0.9461
	2		0.9293	0.9675	0.9691	0.9488
SVM (RBF)	1	0.9475	0.9656	0.9309	0.9271	0.9460
	2		0.9309	0.9656	0.9675	0.9489
LVQ	1	0.9440	0.9675	0.9227	0.9192	0.9427
	2		0.9227	0.9675	0.9689	0.9452
LR	1	0.9406	0.9458	0.9359	0.9306	0.9381
	2		0.9359	0.9458	0.9499	0.9428
DT	1	0.9354	0.9194	0.9530	0.9556	0.9371
	2		0.9530	0.9194	0.9149	0.9336
RF	1	0.9475	0.9309	0.9656	0.9675	0.9489
	2		0.9656	0.9309	0.9271	0.9460

**Table 3:** Classification results for NSGA II selected sets of wavelengths.

Classifier	Class	Accuracy	Sensitivity	Specificity	Precision	F-Score
MLPNN	1	0.9492	0.9638	0.9359	0.9318	0.9476
	2		0.9359	0.9638	0.9660	0.9507
SVM (RBF)	1	0.9483	0.9638	0.9342	0.9302	0.9467
	2		0.9342	0.9638	0.9660	0.9498
LVQ	1	0.9457	0.9566	0.9359	0.9313	0.9438
	2		0.9359	0.9566	0.9595	0.9475
LR	1	0.9156	0.8933	0.9359	0.9268	0.9098
	2		0.9359	0.8933	0.9061	0.9207
DT	1	0.9320	0.9293	0.9349	0.9432	0.9362
	2		0.9349	0.9293	0.9232	0.9290
RF	1	0.9492	0.9675	0.9326	0.9288	0.9477
	2		0.9326	0.9675	0.9692	0.9505

## Data Availability

The data presented in this study are available on request from the corresponding author.

## Abbreviations

The following abbreviations are used in this manuscript:

ACGAN	Auxiliary Classifier Generative Adversarial Network
ANN	Artificial Neural Network
CCD	Charge-Coupled Device
CMOS	Complementary Metal Oxide Semiconductor
CNC	Computer Numerical Control
CNN	Convolutional Neural Network
DNN	Deep Neural Network
DT	Decision Tree
GA	Genetic Algorithm
LB	Laser Beam
LBW	Laser Beam Welding
LVQ	Learning Vector Quantization
LR	Logistic Regression
ML	Machine Learning
MLPNN	Multi-Layer Perceptron Neural Network
NSGA II	Non-dominated Sorting Genetic Algorithm II
PCA	Principal Component Analysis
RBF	Radial Basis Function
RF	Random Forest
ROC	Receiver Operating Characteristic Curve
SOM	Self-Organizing Maps
SVM	Support Vector Machine

## Appendix A

The following equations defines the common indicators that were used to evaluate the performance of the employed models. TP, TN, FP and FN are true positive, true negative, false positive and false negative respectively.

(A1) $$Accuracy=\frac{TP+TN}{TP+TN+FP+FN}$$

(A2) $$Sensitivity=\frac{TP}{TP+FN}$$

(A3) $$Specificity=\frac{TN}{TN+FP}$$

(A4) $$Precision=\frac{TP}{TP+FP}$$

(A5) $$F=2\times \frac{precision\times sensitivity}{precision+sensitivity}$$
